# Impact of light logger and dosimeter placement on wearability and appeal in real-life settings

**DOI:** 10.12688/openreseurope.20985.1

**Published:** 2025-09-22

**Authors:** Johannes Zauner, Anna M. Biller, Manuel Spitschan

**Affiliations:** 1 TUM School of Medicine and Health, Department Health and Sport Sciences, Chronobiology & Health, Technical University of Munich, Munich, Germany; 2 Max Planck Research Group Translational Sensory & Circadian Neuroscience, Max Planck Institute for Biological Cybernetics, Tübingen, Germany; 3 TUM Institute for Advanced Study, Technical University of Munich, Garching, Germany; 4 TUMCREATE Ltd., Singapore, Singapore

**Keywords:** light exposure, wearable light loggers, circadian rhythms, sleep health, visual development, wearability, user-centred design, device placement, human factors, metrology, acceptability, health technology, survey study, daily life contexts, sensor design

## Abstract

**Background:**

Light exposure plays a crucial role in human health, influencing sleep, circadian rhythms, and visual development. As wearable light loggers are increasingly used to monitor real-world light exposure, their design must balance metrological accuracy with comfort and usability.

**Methods:**

We assessed the perceived wearability and appeal of eight light logger designs and placements (chest pin, wrist, necklace, sleeve collar, collar pin, neck loop, hat pin, and glasses) using an online survey completed by 145 participants (mean age 32 years, 52% female) from the UK, USA, and other countries worldwide. Each design was rated on overall attractiveness, likelihood of wear at home, at work, in public settings, in social contexts, during exercise, expected wear duration, and perceived interference with daily activities.

**Results:**

The chest pin received the highest overall ratings, followed by wrist and necklace, while glasses and hat pin placements were rated lowest across most contexts. Likelihood of wear was highest in the home setting and significantly lower in social (β = –0.92 ± 0.08) and work (β = –0.84 ± 0.08) contexts. Compared to the chest pin, other placements were rated up to 2.6 points lower (e.g., glasses: β = –2.62 ± 0.10). Wearing position significantly influenced all ratings (p < 0.001), while sample location and gender showed minimal effects. The thematic analysis of free-text responses revealed concerns around comfort, appearance, stability, and interference with daily activities.

**Conclusions:**

These findings highlight the importance of user-centred design and offer practical guidance for wearable light loggers that are acceptable in everyday contexts.

## Background

Light exposure affects human health and well-being ( Burns *et al*., 2021; Windred *et al*., 2024; Zielinska-Dabkowska *et al*., 2023). Growing evidence has linked light exposure – both daytime and nighttime – to circadian and sleep physiology, resulting to recommendations for optimal light exposure levels ( Brown *et al*., 2022). In addition, exposure to the visual environment, termed the 'visual diet', has been linked to ocular health outcomes such as myopia ( Marcos, 2025). The neurobiological pathways underlying these so-called ‘non-visual’ effects of light – in contrast to the impact of light on the visual system – have been elucidated, involving a particular set of cells in the retina ( Lucas *et al*., 2014), the intrinsically photosensitive retinal ganglion cells (ipRGCs).

Most evidence on non-visual light effects comes from lab studies, but real-world exposure patterns are complex due to behaviour ( Biller *et al*., 2024). Wearable light loggers, sometimes also called dosimeters ^1^, can capture the light exposure of an individual over time ( Danilenko *et al*., 2022; Hartmeyer *et al*., 2022; Spitschan *et al*., 2022). They vary in form, with wrist-worn actimeters being most common and some also capturing light exposure. As the relevant metrological quantity underlying the non-visual effects of light is the activation of the ipRGCs in the retina ( CIE, 2018; Lucas *et al*., 2014; Price & Blattner, 2022; Spitschan, 2019), light exposure should be measured at least in the plane of the eye, which some devices approach by mounting on spectacle frames ( Bierman *et al*., 2005; Hubalek *et al*., 2016; Stampfli *et al*., 2023; Wen *et al*., 2021), with varying acceptability ( Balajadia *et al*., 2023).

As wearable light loggers become central tools in circadian, sleep, and myopia research, understanding user-centered design preferences is key for ensuring high compliance in real-world deployments. To understand the trade-off between the metrological fidelity of light exposure measurements and usability and acceptability in the field, we examined the attractiveness and acceptability of eight light logger designs and placements in a range of contexts in an online survey to guide the development of an optimal form factor of a wearable light logger.

## Methods

### Development of light logger illustrations

**Figure 1.**
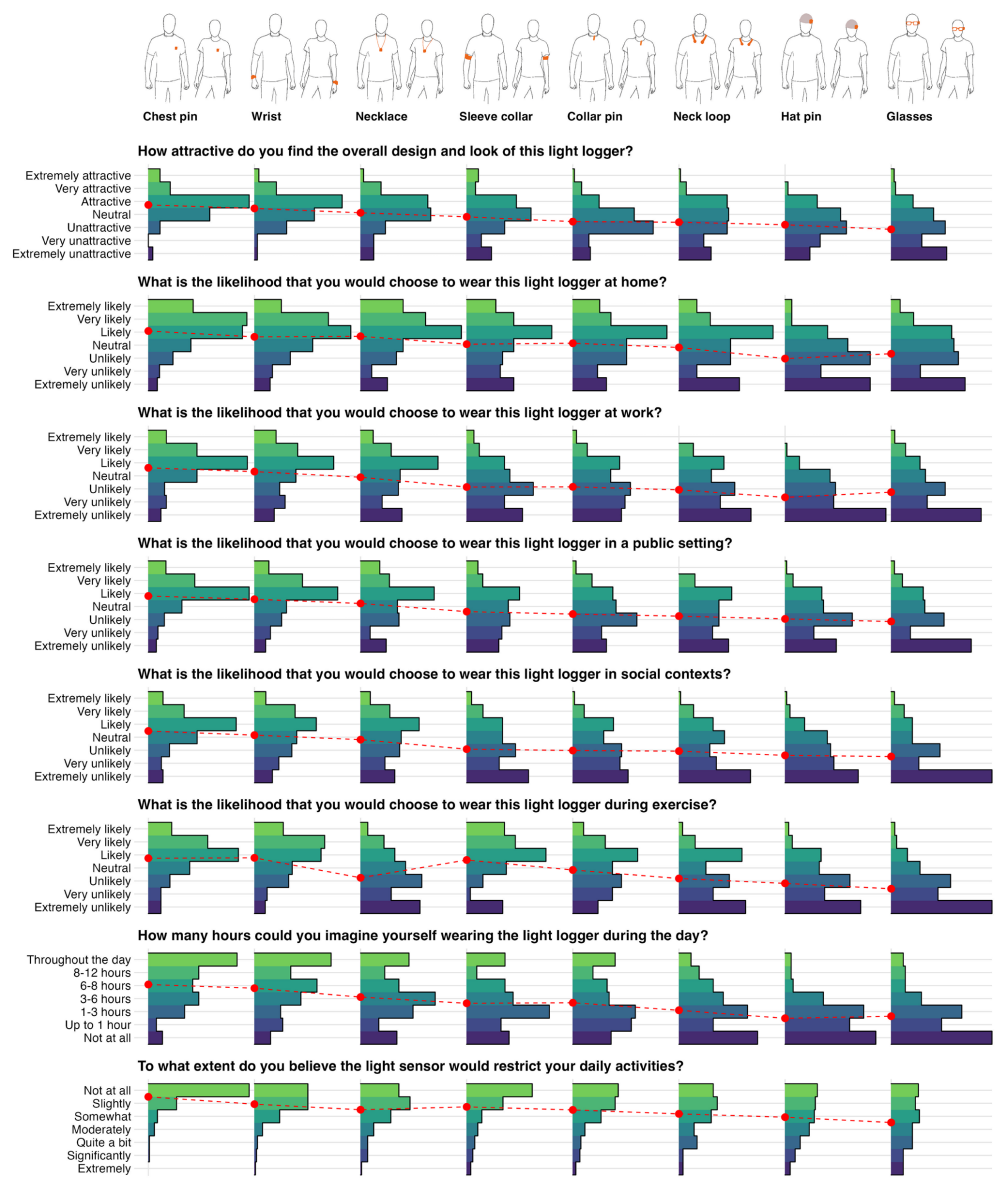
Ratings of light logger wear location. Questions included overall attractiveness and look, different contexts (at home, at work, in a public setting, in social contexts, during exercise), wear duration during the day and interference with daily activities.

Informed by expert domain knowledge, we developed eight possible designs for wearable light loggers: chest pin, wrist, necklace, sleeve collar, collar pin, neck loop, hat pin, and glasses. Some of these designs (chest pin, wrist, necklace, collar pin, and glasses) correspond to existing designs of wearable light loggers, while others were conceived specifically for this study, based on broad knowledge of wearable devices in general. These designs were then turned into 2D illustrations in which a male and female mannequin were shown to wear the designs (see Figure 1, top row).

### Survey design

***Recruitment and procedure.*** We recruited participants through the online platform Prolific ( https://www.prolific.com/). Participants were directed to the survey implemented on REDCap ( Harris *et al*., 2019; Harris *et al*., 2009) (server hosted by the Technical University of Munich), where they gave written informed consent. Recruitment occurred in three waves: US, UK, and worldwide. Participants were compensated at a rate of £3 for an estimated 20 minutes of their time. For each site, a target sample of n=50 was selected due to resource limitations. All data collection took place in January 2024.

***Survey questions.*** We sequentially presented the eight designs and asked a series of rating questions on a 7-item Likert-type scale, probing attractiveness, likelihood to wear the light logger at home, at work, in a public setting, in social contexts, during exercise, how many hours they would wear the device, and to what extent the logger would restrict daily activities. For each design, we also asked the participants the following free-form question: “In what situations or activities do you think wearing the light logger might be difficult?”. We asked participants for the following demographic information: age, sex, gender, native speaker, country of residence, and time zone. The entire survey is available in our GitHub repository ( https://github.com/tscnlab/ZaunerEtAl_OpenResEur_2025/blob/main/data/raw/LightLoggerFormFactorAndPlacem.pdf; also archived on Zenodo) as a PDF printout and as a REDCap data dictionary. For each design, we asked participants to describe the light logger in their own words. Additionally, we added three attention-check questions to avoid the automated completion of questionnaires by bots. We removed all data that failed at least one attention check question.

### Analytic strategy

***Quantitative data.*** Quantitative data were analysed descriptively, inferentially, and visualised using *R* software ( R Core Team, 2017). Inferential analysis was performed using cumulative link mixed models with the *ordinal* package in R ( Christensen, 2019). The dependent variable was the participant's rating. Independent variables included wearing position, sex, the sample (location), and context. Context is a derived variable from survey questions two through six, where all data about the wear-likelihood in different situations is pooled, and the context of each question is stored as a variable. This allows exploration of the wearing position in interaction with the context. Participants’ answers for sex and gender were identical for all participants included in inferential analyses containing those covariates. One participant identified as “Other” for sex and gender and was thus excluded from these analyses, as parameters cannot be estimated for a subgroup containing a single sample. Participants were included as random effect.

*p* values were obtained by likelihood-ratio tests of the full model with the effect in question against the model without the effect in a stepwise elimination of parameters down to the *null model* with only the random effect. *p* values less than or equal to 0.05 were considered significant, with a prior *false discovery rate* (FDR) correction ( Benjamini & Hochberg, 1995). Beta coefficients provide the parameter estimates with the standard error on the logit scale of the model, compared to the reference level, which is the *chest pin* location and *home* context.

Two explorative analyses were performed:

1. How does the wearing position influence each question’s rating, with gender and sample location as control variables? As gender might influence how a given wearing position is rated, the full model includes this interaction effect. Note that sex and gender are identical in our sample. The full model in Wilkinson notation is thus:
Rating∼WearingPosition∗Gender+Sample+(1|Id)(eq 1)
  Post-hoc analyses were performed to identify significantly different wearing positions, also with a *False-Discovery-Rate* correction.
2. How does the context of wear (work, social, etc.) influence the ratings, dependent on the wearing position? The full model in Wilkinson notation is thus:
Rating∼Context∗WearingPosition+(1|Id)(eq 2)
  Gender was not included in this second part for reasons of computational stability, speed, and redundancy with the first explorative analysis, where gender differences are explored within each context.

All scripts used for descriptive and inferential analysis are available as Quarto HTML files in the supporting information.

***Qualitative data.*** Qualitative responses to survey questions were analysed using the large-language model (LLM) Gemini 2.5 Pro Preview 05-06 (Google, Mountain View, USA). The analysis was performed in a three-step process. In the first step, qualitative answers were extracted from the survey and brought into a long format of one answer per question and design per row. In the second step, performed by the LLM, themes were identified, and each participant’s answer in the 16 design-specific questions (8 designs with 2 questions each) and two general questions was assigned to a theme (or sorted to a “non-theme” bin). If multiple themes were touched by a single answer, these answers could be assigned multiple times. These data were exported from the LLM for the final step in R software (see Quarto HTML in the supporting information). In this step, it was checked whether each of the theme-quotes matches a participant’s answer and was not produced through LLM hallucination. This was the case in all quotes. Further, it was checked whether all answers from the survey were part of the thematic analysis, i.e., no answers were left out. This was the case. Finally, multiple rounds of random samples of quotes were manually checked against the themes to check their validity and appropriateness within the theme. Summaries within and across themes were also drafted by the LLM and checked for validity against the dataset.

## Results

### Participant demographics

We were able to recruit a total of 162 participants. One participant did not give their consent and was removed. 16 participants did not pass the attention checks and were removed. One participant did not complete the survey and was removed. The remaining 145 respondents originate from the survey samples as follows: n=47 (UK), n=49 (USA), and n=49 (worldwide). The demographic results for the total sample and the sub-samples are given in Table 1. The average age was 32 years (range 18-81); 75 (52%) were female, 69 were male (48%), and one person identified as 'Other' (0.7%). Most of our participants (95, 66%) were native English speakers, with a larger proportion in the UK and USA samples (85% [UK] and 86% [USA], respectively). In terms of occupation, most participants reported being in full-time employment (46%), followed by people working part-time (19%) or being in education (18%). Our global sample shows a diverse distribution of 17 countries of origin ( Table 2).

**Table 1.** Sample demographics. Answers for gender are identical to sex.

		Sample locations		
Demographics	Overall N = 145 *^1^*	UK N = 47 *^1^*	USA N = 49 *^1^*	Worldwide N = 49 *^1^*
**Age (yrs)**	32 (18–81)	33 (18–75)	39 (19–72)	27 (18–81)
**Sex**				
Female	75 (52%)	26 (55%)	25 (51%)	24 (49%)
Male	69 (48%)	21 (45%)	24 (49%)	24 (49%)
Other	1 (0.7%)	0 (0%)	0 (0%)	1 (2.0%)
**Native English speaker**	95 (66%)	40 (85%)	42 (86%)	13 (27%)
**Occupation**				
In education	26 (18%)	6 (13%)	7 (14%)	13 (27%)
Part-time working	28 (19%)	6 (13%)	14 (29%)	8 (16%)
Full-time working	67 (46%)	26 (55%)	19 (39%)	22 (45%)
Unemployed	17 (12%)	8 (17%)	7 (14%)	2 (4.1%)
Rest	7 (4.8%)	1 (2.1%)	2 (4.1%)	4 (8.2%)

^*1*^Median (Min-Max); n (%)

**Table 2.** Countries of origin of the sample.

**Countries**	N = 145 ^*^1^*^
United States	49 (34%)
United Kingdom	46 (32%)
Italy	10 (6.9%)
Poland	9 (6.2%)
South Africa	9 (6.2%)
Portugal	6 (4.1%)
Spain	3 (2.1%)
Germany	2 (1.4%)
Greece	2 (1.4%)
Hungary	2 (1.4%)
Australia	1 (0.7%)
Belgium	1 (0.7%)
Canada	1 (0.7%)
Czech Republic	1 (0.7%)
Estonia	1 (0.7%)
Ireland	1 (0.7%)
Sweden	1 (0.7%)

^*1*^n (%)

### Attractiveness, usability and acceptability ratings across designs, contexts and samples

***Descriptive analysis.*** The results of this study are shown in Figure 1 and tabulated in Table 3. It is important to note that questions two through six ask about the likelihood of a participant choosing a given design. Therefore, the term likelihood in the results section refers to the phrasing of the question, not the stricter statistical definition.******

**Table 3.** Descriptive statistics of the rating responses.

Item (all N = 145)	Wrist *^1^*	Collar pin *^1^*	Glasses *^1^*	Hat pin *^1^*	Neck loop *^1^*	Chest pin *^1^*	Necklace *^1^*	Sleeve collar *^1^*
**How attractive do you find the overall design and look of this light logger?**								
Extremely unattractive	2 (1.4%)	12 (8.3%)	38 (26%)	17 (12%)	22 (15%)	3 (2.1%)	9 (6.2%)	17 (12%)
Very unattractive	2 (1.4%)	11 (7.6%)	21 (14%)	24 (17%)	17 (12%)	0 (0%)	9 (6.2%)	10 (6.9%)
Unattractive	22 (15%)	55 (38%)	37 (26%)	42 (29%)	33 (23%)	8 (5.5%)	17 (12%)	26 (18%)
Neutral	41 (28%)	42 (29%)	29 (20%)	38 (26%)	34 (23%)	42 (29%)	48 (33%)	44 (30%)
Attractive	60 (41%)	18 (12%)	15 (10%)	22 (15%)	33 (23%)	69 (48%)	46 (32%)	34 (23%)
Very attractive	15 (10%)	6 (4.1%)	3 (2.1%)	2 (1.4%)	5 (3.4%)	15 (10%)	14 (9.7%)	6 (4.1%)
Extremely attractive	3 (2.1%)	1 (0.7%)	2 (1.4%)	0 (0%)	1 (0.7%)	8 (5.5%)	2 (1.4%)	8 (5.5%)
**What is the likelihood that you would choose to wear this light logger in a public setting?**								
Extremely unlikely	7 (4.8%)	21 (14%)	50 (34%)	32 (22%)	31 (21%)	5 (3.4%)	16 (11%)	20 (14%)
Very unlikely	10 (6.9%)	18 (12%)	12 (8.3%)	18 (12%)	21 (14%)	6 (4.1%)	6 (4.1%)	22 (15%)
Unlikely	17 (12%)	40 (28%)	33 (23%)	42 (29%)	25 (17%)	10 (6.9%)	24 (17%)	27 (19%)
Neutral	20 (14%)	27 (19%)	21 (14%)	24 (17%)	25 (17%)	21 (14%)	23 (16%)	26 (18%)
Likely	52 (36%)	25 (17%)	20 (14%)	23 (16%)	33 (23%)	63 (43%)	46 (32%)	33 (23%)
Very likely	30 (21%)	12 (8.3%)	7 (4.8%)	5 (3.4%)	10 (6.9%)	29 (20%)	18 (12%)	10 (6.9%)
Extremely likely	9 (6.2%)	2 (1.4%)	2 (1.4%)	1 (0.7%)	0 (0%)	11 (7.6%)	12 (8.3%)	7 (4.8%)
**What is the likelihood that you would choose to wear this light logger at home?**								
Extremely unlikely	7 (4.8%)	15 (10%)	33 (23%)	38 (26%)	27 (19%)	4 (2.8%)	12 (8.3%)	21 (14%)
Very unlikely	8 (5.5%)	11 (7.6%)	13 (9.0%)	16 (11%)	8 (5.5%)	5 (3.4%)	5 (3.4%)	15 (10%)
Unlikely	16 (11%)	24 (17%)	30 (21%)	38 (26%)	23 (16%)	11 (7.6%)	16 (11%)	16 (11%)
Neutral	26 (18%)	24 (17%)	28 (19%)	28 (19%)	23 (16%)	19 (13%)	19 (13%)	21 (14%)
Likely	43 (30%)	42 (29%)	27 (19%)	19 (13%)	42 (29%)	42 (29%)	45 (31%)	38 (26%)
Very likely	33 (23%)	17 (12%)	10 (6.9%)	3 (2.1%)	14 (9.7%)	44 (30%)	29 (20%)	21 (14%)
Extremely likely	12 (8.3%)	12 (8.3%)	4 (2.8%)	3 (2.1%)	8 (5.5%)	20 (14%)	19 (13%)	13 (9.0%)
**What is the likelihood that you would choose to wear this light logger at work?**								
Extremely unlikely	11 (7.6%)	27 (19%)	50 (34%)	56 (39%)	40 (28%)	7 (4.8%)	23 (16%)	31 (21%)
Very unlikely	17 (12%)	29 (20%)	20 (14%)	27 (19%)	23 (16%)	10 (6.9%)	12 (8.3%)	21 (14%)
Unlikely	14 (9.7%)	32 (22%)	30 (21%)	28 (19%)	31 (21%)	9 (6.2%)	21 (14%)	37 (26%)
Neutral	23 (16%)	21 (14%)	19 (13%)	25 (17%)	18 (12%)	27 (19%)	22 (15%)	24 (17%)
Likely	44 (30%)	26 (18%)	16 (11%)	8 (5.5%)	25 (17%)	55 (38%)	43 (30%)	21 (14%)
Very likely	24 (17%)	8 (5.5%)	8 (5.5%)	1 (0.7%)	8 (5.5%)	27 (19%)	17 (12%)	7 (4.8%)
Extremely likely	12 (8.3%)	2 (1.4%)	2 (1.4%)	0 (0%)	0 (0%)	10 (6.9%)	7 (4.8%)	4 (2.8%)
**What is the likelihood that you would choose to wear this light logger in social contexts?**								
Extremely unlikely	11 (7.6%)	34 (23%)	62 (43%)	45 (31%)	44 (30%)	9 (6.2%)	21 (14%)	38 (26%)
Very unlikely	15 (10%)	29 (20%)	17 (12%)	30 (21%)	23 (16%)	8 (5.5%)	15 (10%)	20 (14%)
Unlikely	23 (16%)	30 (21%)	30 (21%)	28 (19%)	19 (13%)	13 (9.0%)	24 (17%)	30 (21%)
Neutral	26 (18%)	19 (13%)	13 (9.0%)	26 (18%)	28 (19%)	30 (21%)	25 (17%)	22 (15%)
Likely	38 (26%)	25 (17%)	13 (9.0%)	12 (8.3%)	21 (14%)	54 (37%)	36 (25%)	22 (15%)
Very likely	25 (17%)	7 (4.8%)	8 (5.5%)	3 (2.1%)	9 (6.2%)	22 (15%)	18 (12%)	10 (6.9%)
Extremely likely	7 (4.8%)	1 (0.7%)	2 (1.4%)	1 (0.7%)	1 (0.7%)	9 (6.2%)	6 (4.1%)	3 (2.1%)
**What is the likelihood that you would choose to wear this light logger during exercise?**								
Extremely unlikely	6 (4.1%)	14 (9.7%)	56 (39%)	42 (29%)	37 (26%)	7 (4.8%)	33 (23%)	20 (14%)
Very unlikely	7 (4.8%)	22 (15%)	26 (18%)	22 (15%)	19 (13%)	7 (4.8%)	17 (12%)	2 (1.4%)
Unlikely	19 (13%)	27 (19%)	33 (23%)	36 (25%)	28 (19%)	12 (8.3%)	34 (23%)	9 (6.2%)
Neutral	21 (14%)	22 (15%)	16 (11%)	19 (13%)	15 (10%)	23 (16%)	25 (17%)	22 (15%)
Likely	37 (26%)	36 (25%)	9 (6.2%)	20 (14%)	35 (24%)	50 (34%)	19 (13%)	44 (30%)
Very likely	39 (27%)	18 (12%)	3 (2.1%)	4 (2.8%)	9 (6.2%)	33 (23%)	13 (9.0%)	27 (19%)
Extremely likely	16 (11%)	6 (4.1%)	2 (1.4%)	2 (1.4%)	2 (1.4%)	13 (9.0%)	4 (2.8%)	21 (14%)
**How many hours could you imagine yourself wearing the light logger during the day?**								
Not at all	8 (5.5%)	19 (13%)	50 (34%)	45 (31%)	39 (27%)	7 (4.8%)	18 (12%)	21 (14%)
Up to 1 hour	14 (9.7%)	29 (20%)	25 (17%)	32 (22%)	17 (12%)	4 (2.8%)	9 (6.2%)	17 (12%)
1-3 hours	13 (9.0%)	31 (21%)	35 (24%)	39 (27%)	34 (23%)	18 (12%)	26 (18%)	41 (28%)
3-6 hours	23 (16%)	18 (12%)	15 (10%)	19 (13%)	22 (15%)	25 (17%)	37 (26%)	23 (16%)
6-8 hours	31 (21%)	17 (12%)	7 (4.8%)	4 (2.8%)	17 (12%)	22 (15%)	19 (13%)	19 (13%)
8-12 hours	18 (12%)	10 (6.9%)	7 (4.8%)	3 (2.1%)	10 (6.9%)	25 (17%)	12 (8.3%)	5 (3.4%)
Throughout the day	38 (26%)	21 (14%)	6 (4.1%)	3 (2.1%)	6 (4.1%)	44 (30%)	24 (17%)	19 (13%)
**To what extent do you believe the light sensor would restrict your daily activities?**								
Not at all	53 (37%)	45 (31%)	27 (19%)	32 (22%)	34 (23%)	100 (69%)	38 (26%)	65 (45%)
Slightly	53 (37%)	42 (29%)	24 (17%)	30 (21%)	38 (26%)	28 (19%)	49 (34%)	36 (25%)
Somewhat	25 (17%)	28 (19%)	28 (19%)	29 (20%)	32 (22%)	9 (6.2%)	30 (21%)	15 (10%)
Moderately	8 (5.5%)	12 (8.3%)	21 (14%)	24 (17%)	15 (10%)	6 (4.1%)	13 (9.0%)	11 (7.6%)
Quite a bit	3 (2.1%)	8 (5.5%)	21 (14%)	15 (10%)	18 (12%)	1 (0.7%)	7 (4.8%)	8 (5.5%)
Significantly	2 (1.4%)	7 (4.8%)	12 (8.3%)	10 (6.9%)	4 (2.8%)	1 (0.7%)	7 (4.8%)	6 (4.1%)
Extremely	1 (0.7%)	3 (2.1%)	12 (8.3%)	5 (3.4%)	4 (2.8%)	0 (0%)	1 (0.7%)	4 (2.8%)

^*1*^n (%)

Overall, the *Chest pin* position was rated most favorable, followed by *Wrist*, *Necklace*, *Sleeve collar*, *Collar pin*, *Neck loop*, *Hat pin*, and, least favorable, *Glasses*. The order from best to lowest ranking is:

1. Chest pin
2. Wrist
3. Necklace
4. Sleeve collar
5. Collar pin
6. Neck loop
7. Hat pin
8. Glasses

This order holds across all questions, with very few exceptions. Notably, people rated the *Necklace* position less favorable for exercise than the *Sleeve collar* or *Collar pin,* and rated the *Necklace* as slightly more restricting than the *Sleeve collar* during daily activities. Further, the *Hat pin* position was rated lower than *Glasses* for wear at home and work.

**Figure 2.**
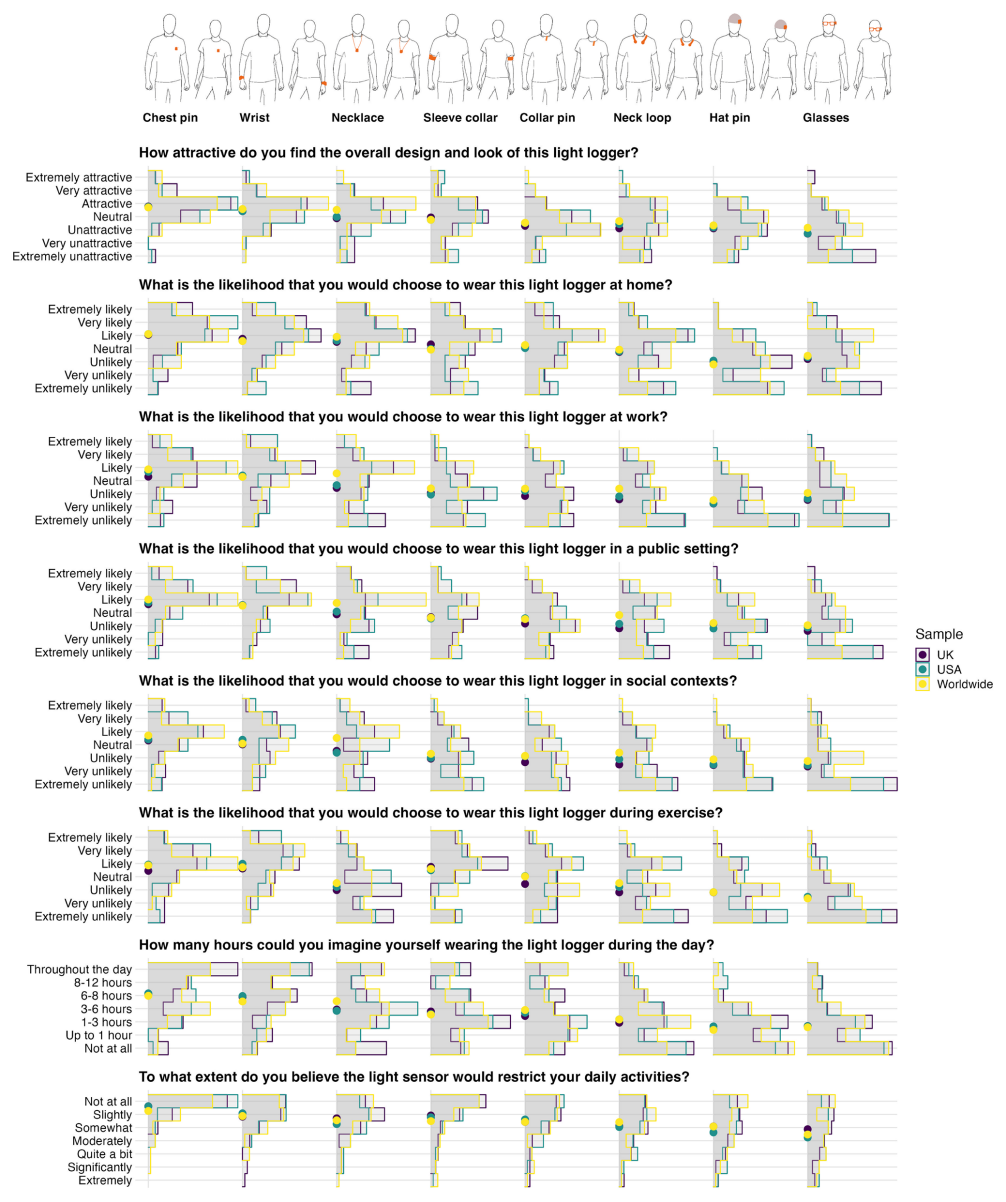
Light logger wear location ratings by sample locations.

**Figure 3.**
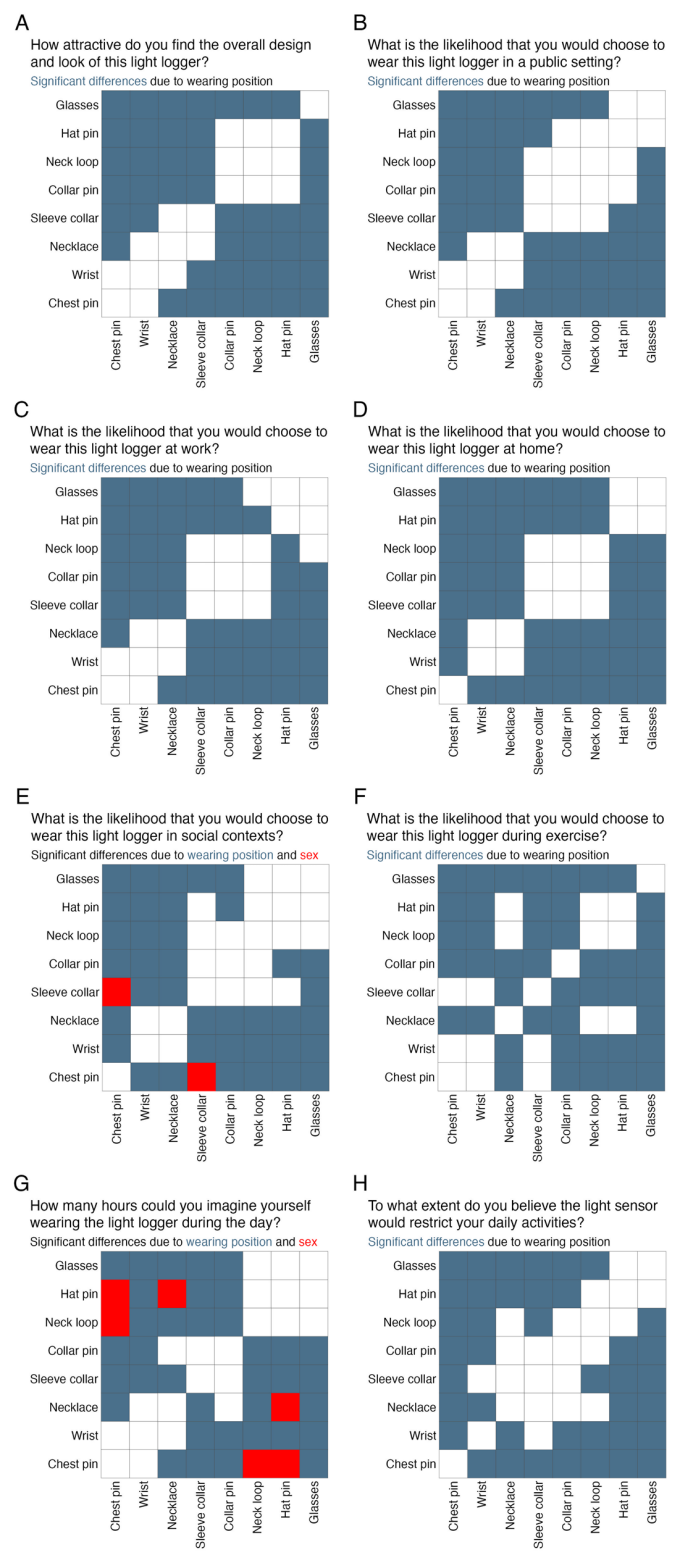
Statistically significant differences in ratings between wearing position pairs. Blue squares indicate that ratings differ significantly between these pairs, and red squares further indicate a significant difference in rating depending on sex/gender for these pairs. Note that participants’ answers for sex and gender were identical in our sample.

***Inferential analysis.*** The first branch of the exploratory analysis examined how the wearing position influences each question’s rating, with sex/gender and sample location as control variables. The wearing position contributes significantly to explain ratings in all questions (all p < 0.001), with the basic order being the same as described in the descriptive analysis results above. Figure 3 shows which wearing positions differ significantly from one another in their rating. Sample location had no effect (all p ≥ 0.077; Figure 2), and neither did gender (all p ≥ 0.61). The interaction of gender and wearing position was significant in the questions about the social context (p = 0.032) and for the wearing duration (p = 0.005). In both cases, the interaction significantly affected only a few wearing positions. Namely, in a social context, male participants rated the *Sleeve collar* position about one step better than female participants ( *β* = 1.2 ± 0.43), leading to about the same rating as the *Necklace* position. For wear duration, men would be willing to wear the *Neck loop* ( *β* = 1.33 ± 0.44) or the *Hat pin* ( *β* = 1.51 ± 0.44) about one to one and a half steps longer than females. Figure 3 shows where the interaction of sex and wearing position leads to further significant differences in rating compared to the wearing position alone.

The second branch of the exploratory analysis examined how context influences the ratings. This analysis thus excluded the questions on attractiveness, wearing duration, and restriction, and only included the questions about the likelihood of choosing to wear a particular design in a public setting, at work, at home, in social contexts, or during exercise. In this model, context, wearing position, and their interaction were significant (all p < 0.001). Context significantly influenced ratings: participants rated designs more favorably at home than in public ( *β* = −0.45 ± 0.07), during exercise ( *β* = −0.45 ± 0.08), at work ( *β* = −0.84 ± 0.08), or in social setting ( *β* = −0.92 ± 0.08), where a *β* of about −1 equals one rating level lower (less favorable). Table 4 shows the likelihood of wear ratings depending on context. For wearing position, people are more willing to rate the *Chest pin* position higher (more favorable) regardless of context, compared to *Wrist* ( *β* = −0.34 ± 0.09), *Necklace* ( *β* = −1.02 ± 0.09), *Sleeve collar* ( *β* = −1.40 ± 0.10), *Collar pin* ( *β* = −1.62 ± 0.10), *Neck loop* ( *β* = −1.99 ± 0.10), *Hat pin* ( *β* = −2.60 ± 0.10), or *Glasses* position ( *β* = −2.62 ± 0.10), where a *β* of about –1 equals one rating level lower (less favourable). Table 5 shows the likelihood of wear ratings depending on wearing position. Looking at the combined effect of context and wearing position, Table 6 shows the predicted mode rating of each wearing position depending on context. The table summarises concisely that the *Chest pin* and *Wrist* are generally accepted wearing positions (mode: *likely*). The *Necklace* is mostly accepted but rated *unlikely* for exercise. *Sleeve collar*, *Collar pin*, and *Neck loop* are accepted for the home context and are partly preferable for exercise but are rated *unlikely* for other contexts. The *Hat pin* and *Glasses* are the lowest rated wearing positions overall and are rated *extremely unlikely* to be worn in a work or social setting.

**Table 4.** Model prediction for the likelihood of wear ratings depending on context. The colouring emphasises the range of numeric values.

Parameters	Extremely unlikely	Very unlikely	Unlikely	Neutral	Likely	Very likely	Extremely likely
home	8%	9%	17%	19%	30%	13%	4%
public	13%	12%	20%	19%	25%	9%	3%
exercise	13%	12%	20%	19%	25%	9%	3%
work	18%	14%	22%	18%	20%	6%	2%
social	19%	15%	23%	17%	19%	6%	2%

**Table 5.** Model prediction for the likelihood of wear ratings depending on wearing position. The colouring emphasises the range of numeric values.

Parameters	Extremely unlikely	Very unlikely	Unlikely	Neutral	Likely	Very likely	Extremely likely
Chest pin	3%	4%	10%	16%	39%	21%	7%
Wrist	4%	5%	13%	19%	37%	17%	5%
Necklace	8%	9%	19%	22%	30%	10%	3%
Sleeve collar	11%	12%	23%	22%	25%	7%	2%
Collar pin	13%	13%	24%	21%	21%	6%	1%
Neck loop	18%	16%	25%	19%	17%	4%	1%
Hat pin	28%	20%	24%	14%	10%	2%	1%
Glasses	29%	21%	24%	14%	10%	2%	1%

**Table 6.** Model prediction for the mode rating depending on context and wearing position. The colouring emphasises the range of the rating.

Context	Chest pin	Wrist	Necklace	Sleeve collar	Collar pin	Neck loop	Hat pin	Glasses
home	Likely	Likely	Likely	Likely	Likely	Likely	Unlikely	Unlikely
public	Likely	Likely	Likely	Unlikely	Unlikely	Unlikely	Unlikely	Extremely unlikely
exercise	Likely	Likely	Unlikely	Likely	Likely	Unlikely	Unlikely	Extremely unlikely
work	Likely	Likely	Likely	Unlikely	Unlikely	Unlikely	Extremely unlikely	Extremely unlikely
social	Likely	Likely	Likely	Unlikely	Unlikely	Unlikely	Extremely unlikely	Extremely unlikely

***Qualitative results.*** The analysis identified three overarching themes and several specific sub-themes influencing the wearability and acceptability of the light logger designs. All themes and the number of instances these were mentioned by users can be found in Table 7.

**Table 7.** Thematic analysis. Values show number of participant-replies per theme.

		General	Chest pin	Wrist	Necklace	Sleeve collar	Collar pin	Neck loop	Hatpin	Glasses
Alternative suggestions	Participant ideas for other designs or improvements.	142	---	---	---	---	---	---	---	---
Miscellaneous comments	Miscellaneous comments about the survey or logger.	111	---	---	---	---	---	---	---	---
Body placement perception	General descriptions or opinions on the specific body location.	---	149	145	142	140	151	139	140	140
Comfort and discomfort: Concerns about the physical experience of wearing the logger (e.g. irritation, weight, bulk).										
Comfort physical	Physical sensations like irritation, weight, tightness, bulkiness, heat.	1	6	12	6	21	10	32	19	16
Social and appearance: Concerns related to social perception, appearance, and appropriateness of the logger.										
Social acceptability	Concerns about appearance, looking odd, conspicuousness, or professionalism.	3	20	15	14	31	57	33	27	36
Privacy perception	Concerns that the logger might be mistaken for a recording device.	---	2	---	---	---	3	---	1	7
Situational appropriateness	Whether the design is suitable for specific contexts beyond just social (e.g., formal events, indoor vs. outdoor).	---	8	8	4	8	6	2	55	5
Practicality and usability: General usability and practicality concerns across designs.										
Activity interference	The logger hindering specific physical activities (work, sports, chores).	---	26	64	88	34	55	65	33	58
Attachment security	Worries about the logger falling off or not being secure.	---	6	---	1	1	11	17	3	2
Clothing integration	How logger interacts with clothing (e.g., being covered by layers, damaging clothes, needing specific attire).	---	4	---	---	8	1	1	---	---
Clothing integration	How the logger interacts with clothing (e.g., being covered by layers, damaging clothes, needing specific attire).	---	25	2	6	30	15	6	2	1
Durability	Concerns about the logger breaking or being damaged.	---	2	2	1	1	2	1	1	1
Ease of use	Practical aspects like forgetting to put it on, switching between devices.	---	5	---	---	---	2	3	1	2
Sleep interference	Specific concerns about the logger being difficult or uncomfortable to wear while sleeping.	---	5	13	6	6	6	7	5	11
Water resistance	Concerns about water exposure (showering, swimming, rain, dishes).	---	13	56	15	15	15	15	8	23
Preexisting habits	How the logger fits with existing habits (e.g., not wearing hats/glasses, already wearing a watch).	---	---	5	1	2	---	1	50	45
Restriction of movement	General feeling of being restricted, not tied to a specific activity.	---	---	1	---	3	---	5	---	2

A complete list of participant answers sorted into themes can be found at: https://github.com/tscnlab/ZaunerEtAl_OpenResEur_2025/blob/main/data/cleaned/qual_analysis_LLM.txt

### Practicality & usability

This was a dominant theme, reflecting concerns about how well the logger would function in daily life.

- **Activity interference:** A major concern across almost all designs. Participants frequently cited difficulties wearing loggers during physical work (e.g., "I work for a moving company"), exercise ("intense exercise", "rock climbing", "running", "lifting weights"), or daily chores ("cooking", "doing dishes").
  • *Wrist:* "my main hobby is rock climbing", "typing or using a computer".
  • *Collar pin, Chest pin, Necklace:* Concerns about them falling off or hindering movement during "intense exercise".
  • *Glasses:* "working out", "anything with a lot of movement".
  • *Neck loop:* "Anything with a lot of movement", "Doing exercises".
- **Sleep interference:** A frequently cited concern for nearly all logger designs was the anticipated difficulty or discomfort of wearing them during sleep. Participants explicitly mentioned "Sleep hours" as problematic for designs like the *Collar pin, Glasses, Hat pin, Neck loop, Chest pin, Necklace,* and *Sleeve collar*. The *wrist* logger also prompted comments such as, "light logger might be difficult wearing when sleeping. Would not be able to sleep on my hand properly", and general difficulty "when sleeping or laying in bed". For the *glasses* design, one participant stated, "I would not wear it to sleep". The *Hat pin* was also singled out: "Exercising, sleeping, taking a shower, swimming will be difficult to do". This indicates that achieving continuous wear through the night presents a significant design challenge across most form factors, as users prioritise comfort and unhindered rest.
- **Attachment security:** Primarily for pin/clip-based designs ( *collar pin*, *chest pin*) and loose-fitting designs ( *neck loop*, *Necklace*). Participants worried about devices "falling off easily" or not being "stable".
- **Clothing integration:** How the logger interacts with clothing was a common point.
  • *Wrist, sleeve collar:* Obstruction by "long sleeved shirt or jacket", "cold climates where you wear lots of layers",
  • *Chest pin, collar pin:* Being covered by a "coat", or issues with "different shaped clothing". Some worried about pins "damaging clothing".
- **Water resistance:** Frequently mentioned for designs in direct skin contact or likely to get wet. "Showering/bathing", "swimming", "rainy weather", and "doing dishes" were common examples where participants would be concerned or remove the device. This applied strongly to *Wrist, Glasses, Hat pin, Neck loop, Chest pin, Necklace, Sleeve collar*.
- **Durability:** Some participants, especially for the *Wrist* design, drew parallels to other wearables, expressing concerns like, "I used to wear an apple watch that I destroyed pretty quickly".
- **Ease of use & pre-existing habits:**
  • *Glasses:* "If you do not already wear glasses it forces you to", "having to switch out" with regular glasses.
  • *Hat pin:* "I rarely wear hats", "any work setting or anywhere it would be uncomfortable / unfit to wear a beanie".
  • *Wrist:* For some, if it's "not also a watch, I would likely find it uncomfortable to wear a watch like device on both arms".
  • Forgetting to transfer pin/clip devices when changing clothes was also a minor ease-of-use concern.

### Social & appearance concerns

The aesthetic and social perception of the loggers significantly influenced acceptability.

- **Social acceptability:** Many designs were deemed "conspicuous", "silly", "ugly", "awkward", or "unfashionable", particularly for "social settings", "formal events", or "work".
  • *Collar pin:* "Looks like a bad/conspicuous spot so wearing it socially wouldn't be ideal."
  • *Glasses:* "Attention drawn to my face".
  • *Hat pin:* "Unfashionable in work and social situations".
  • *Neck-loop:* "Ugly", "silly in my opinion".
  • *Sleeve-collar:* "Looks like exercise or medical equipment".
- **Privacy Perception:** Some designs, notably *Glasses*, *chest pin*, and *collar pin*, raised concerns about being mistaken for a "hidden camera or recording devices", making others uncomfortable.
- **Situational appropriateness:** Beyond general social settings, some designs were seen as inappropriate for specific contexts.
  • *Hat pin:* "Indoors - where hats are uncommon", "not polite".
  • *Chest pin/Collar pin:* Concerns about looking "unprofessional" or drawing unwanted questions.

### Physical comfort & discomfort

- **Comfort/physical sensation:** Issues included potential "skin irritation", "extra weight" (especially for *Glasses*), feeling "clunky", "bulky", "heavy", or generally "uncomfortable".
  • *Wrist:* "Irritated by texture/weight on your wrist".
  • *Collar pin:* "Seems uncomfortable".
  • *Neck loop:* "Irritating to have some weight / thing hang around your neck".
  • *Necklace:* "Annoying [...] something rubbing against your neck".
  • *Sleeve collar:* "Irritating on my upper arm".
  • *Hat pin:* Hat itself becoming "hot" or "messing up hair".

### Design-specific perceptions & alternative suggestions

- **Wrist:** Often seen as the most familiar ("like a watch", "Fitbit type"), but concerns about bulkiness, water, and interference with tasks/other watches existed.
- **Collar pin & Chest pin:** Liked by some for potential discreetness if small, but others found the placement odd, worried about security, clothing damage, or looking like a microphone. The *chest-pin* design can be inconspicuous ("Doesn't appear as though it would interfere").
- **Glasses:** Concerns included weight, bulk, peripheral vision, and social awkwardness. A potential issue are wearers who don’t normally wear glasses.
- **Hat pin:** Limited by the necessity of wearing a hat, making it unsuitable for many situations (indoors, warm weather, formal settings).
- **Neck loop:** Frequently criticised as "ugly", "silly", "cumbersome", and prone to falling off.
- **Necklace:** Seen as potentially acceptable if discreet, but concerns about swinging during activity and tangling were common.
- **Sleeve collar:** Mixed reactions; some saw it as non-intrusive, others as restrictive, uncomfortable, or socially awkward ("weird arm band").
- **Alternative suggestions:** A strong recurring suggestion was for **smaller, more discreet designs**. Specifically, a **ring** was mentioned by some participants ("maybe a ring because a lot of people already wear rings," "a ring version [...] low-key"). **Earrings** or **hair clips/accessories** were also suggested, aiming for fashion integration. Integration into existing clothing ("fabric integration", "stickers") or multi-functional devices (e.g., combined with a smartwatch) were other ideas. Some suggested a "small clip (the size of a stamp) that can be attached anywhere".

## Discussion

### Differences in attractiveness, usability and acceptability in light logger design and placement

Our study revealed that there are marked differences in the subjective prospective attractiveness, usability and acceptability of wearable light loggers as assessed in a survey based on 2D design prototypes. We found clear differences in these properties across device design and placement and between contexts. The findings suggest that in order to optimise wearable light loggers for field use and translational applications, special care must be taken when selecting a specific wearable design. When comparing ratings, the *Chest pin* design was most favoured by participants, followed by *Wrist*, *Necklace*, and *Sleeve collar.* The least favoured was the *Glasses* design, followed by *Hat pin*, *Neck loop*, and *Collar pin*. This order was very stable across situations, with only slight deviations connected to exercise (where the loose *Necklace* design was rated worse than the *Sleeve collar* and *Collar pin*) or gender (where the *Sleeve collar* position was rated better by male participants in a social context). Notably, even the best-rated design, the *Chest pin* raised concerns regarding conspicuity, physical discomfort, and suitability for different clothing types. Looking at qualitative feedback, the findings highlight a clear tension between the need for a light logger to be exposed to ambient light and the user’s desire for discretion, comfort, and seamless integration into daily life. Practicality concerns, such as interference with activities and water resistance, are paramount. Social acceptability and physical comfort are also major determinants of willingness to wear a device continuously. No single design was universally accepted, but those resembling familiar items (wrist-watch) or offering high potential for discretion (a very small chest-pin or a sleek necklace) were generally preferred over obtrusive or habit-changing designs (glasses for non-wearers, hat-pins, neck-loops). The strong preference for miniaturised and integrated alternatives (rings, earrings) suggests that future light logger development should prioritise unobtrusiveness and aesthetic appeal, potentially through co-design with users. Addressing concerns about activity interference, attachment security, and water resistance will also be critical for achieving high wearability and compliance in research settings.

### Use of subjective measures

We probed the attractiveness, usability and acceptability using subjective ratings based on 2D illustrations. This approach allows for probing preferences but does not replace user testing through physical devices. Future research needs to examine the usability of different physical light logger designs, e.g., through non-functional prototypes as a starting point, through qualitative interviews, focus groups, and field testing with both short and longitudinal wear time. Data from our survey-based approach could then be compared and confirmed, or disconfirmed, to the empirical data collected in such an approach. As wearable light loggers continue to be developed and receive attention also in clinical contexts, such studies will become of increasing importance.

### Single-context evaluation

In this study, we asked participants to evaluate the wearable light logger designs and placement locations across a series of contexts, namely at home, at work, in public setting, in social contexts, and during exercise. However, even within these ‘macro-contexts’, there may be significant differences in the appropriateness of different designs and placements. For example, swimming or underwater exercise certainly falls into the category of ‘exercise’, but research-grade wearable light loggers can typically not be used in these contexts. A more fine-grained evaluation of different activities may be necessary to understand differences in different types of exercise or physical activities (e.g., running vs. yoga). Similarly, it may be the case that for light loggers only worn in occupational settings, some specific designs will be more appropriate than others. Season might be a major additional factor, as clothing and clothing-related behaviour changes quite strongly between winter and summer. Additionally, the length during which these designs are worn (not just the hours in the day, which was asked in the survey) could change people’s perception of the designs.

### Population-specific considerations

As our sample included only adults, our findings may not extend to other populations. Children are likely to have different needs and preferences when it comes to wearable devices, influenced by factors like body size, clothing, and daily routines. The same holds for other groups, such as older adults or people in different cultural or occupational contexts. These differences underline the importance of considering the specific needs of each target group when developing wearable devices. Ideally, this should be done in collaboration with the people who will actually use them through co-design and co-creation approaches.

## Conclusion

In this study, we examined subjective usability and acceptability of eight distinct designs and placement locations for wearable light loggers. Our results show marked differences in attractiveness, usability, and acceptability across light logger designs. We did not see differences based on the location of the participants (UK, USA, worldwide). Future research needs to be done to examine behavioural compliance with different physical light logger designs and placement locations to optimise the trade-off between metrological fidelity and use of wearable light loggers in field and translational contexts.

## Statements

### Ethical standards

This study was reviewed and approved by the TUM Ethics Committee (2024-26-W-CB, 23 January 2024). Participants provided written informed consent to participate in the study.

## Data Availability

Zenodo. *Zauner *et al*. Open Research Europe 2025 Dataset.* https://doi.org/10.5281/zenodo.16638151. ( Zauner *et al.*, 2025) This project contains the following underlying data: LightLoggerFormFacto_CLEANED_DATA_2024-02-19.csv. (Survey responses.) Data is available under the terms of the Creative Commons Attribution 4.0 International license (CC BY 4.0).

## Software availability statement

- GitHub. *Zauner *et al*. Open Research Europe 2025 Software Repository.* **https://github.com/tscnlab/ZaunerEtAl_OpenResEur_2025.**
  Zenodo. *Archived source code at time of publication.* https://doi.org/10.5281/zenodo.16779274.
  This project contains the following underlying software:
  Source code. (R scripts and documentation for data processing and analysis.)
  Software is available under the terms of the MIT License.
