# Supplementary material for: Impact of light logger and dosimeter placement on wearability and appeal in real-life settings
Source: Open Res Eur. 2025 Sep 22;5:293. [Version 1] doi: 10.12688/openreseurope.20985.1 (PMC12514442; doi:10.12688/openreseurope.20985.1)
Supplement: Peer review history

# Reviewer report: Reviewer response for version 1

**DOI:** 10.21956/openreseurope.22703.r61075

**Published:** 2025-10-11

**Authors:** Sietse de Vries

**Affiliations:** 1 Eindhoven University of Technology, Eindhoven, The Netherlands

The article presents findings from an online survey on the perceived user attractiveness and acceptability of various light logger designs and positions across different contexts. As light loggers are essential tools for characterizing personal light exposure in real-world settings, these findings are highly relevant for study design. Selecting a wear position with higher attractiveness/acceptability is likely to improve participant compliance and minimize changes in participant behavior as a result of wearing the light logger, resulting in higher data quality.  Given the growing need for field studies on personal light exposure to investigate non image- forming effects and myopia development, methodological guidance of this kind is timely and relevant. Although the results are based on a survey with 2D images, and the authors rightly call for follow-up studies that more closely approximate real-world use cases, the findings provide a valuable starting point for light logger designs that are broadly acceptable to most participants.

 The analysis appears to be well executed. Although I do not possess the statistical expertise to fully evaluate the specific tests used, they appear to be appropriate and well-applied. The article is well structured and mostly clearly presented. Below are some suggestions for improvement.

- Introduction, page 4. The need for information on light logger design preferences to improve user compliance is described. The subsequent sentence introduces the tradeoff between metrological fidelity and attractiveness/acceptability. However, it is not clear whether metrological fidelity refers solely to user compliance or also to the validity of (eye-level) light exposure measurements when the light logger is worn on the body, i.e., away from the eye. Clarifying this point would strengthen the argument for this study: if metrological fidelity consists of user compliance and measurement validity, then selecting the appropriate tradeoff is even more critical.
- Figures 1 and 2. It would be helpful if the captions explained what the dots represent (median values?). In Figure 1, while the dashed line between the dots makes it easier to see how scores deviate between wear locations, it is not entirely appropriate, as the data is categorical. Additionally, the way the data distributions are visualized is not clear. The same number of responses in Table 3 does not seem to correspond to bars of equal length, which could potentially be misleading. Clarification on how these bars are scaled would improve interpretability. For example, by including a horizontal axis at the bottom of both figures, indicating the values (i.e., absolute numbers or percentages).
- Methods, page 4. It is stated that the eight designs were presented sequentially. Was this sequence randomized? If this is not the case, could the order have influenced the participants’ responses? For example, a chest pin might be rated more positively if it was shown immediately after the device on glasses. It would be helpful to clarify this.
- Results, page 6. It is stated that “our global sample shows a diverse distribution of 17 countries of origin.” While including participants from 17 countries is commendable, apart from 9 participants from South Africa and 1 from Australia, all participants were from European or North American countries. I suggest clarifying this in the text. This would align with the point already raised in the discussion (page 17) that the findings may not be representative of people in different cultural contexts.
- Results, page 6. The order of the most favorably rated positions is first given in text, and subsequently as a list. Consider removing the repetition for conciseness.
- Methods, page 6. The Wilkinson notation is described. There appears to be an encoding error (in the PDF download) in both notations (□).
- Conclusion, page 16. Consider mentioning the main findings from the survey; the most preferred wear position.
- Statements, page 16. Ethical approval for the study is stated to have been granted on January 23. On page 4, it is mentioned that all data collection took place in January. Please clarify the timeline.

Is the study design appropriate and does the work have academic merit?

Yes

Is the work clearly and accurately presented and does it cite the current literature?

Yes

If applicable, is the statistical analysis and its interpretation appropriate?

Yes

Are all the source data underlying the results available to ensure full reproducibility?

Yes

Are the conclusions drawn adequately supported by the results?

Yes

Are sufficient details of methods and analysis provided to allow replication by others?

Yes

Reviewer Expertise:

Lighting simulation, light-dosimetry methodology, light sensor optimization, integrative lighting

I confirm that I have read this submission and believe that I have an appropriate level of expertise to confirm that it is of an acceptable scientific standard.

# Reviewer report: Reviewer response for version 1

**DOI:** 10.21956/openreseurope.22703.r61079

**Published:** 2025-10-11

**Authors:** Myriam Aries

**Affiliations:** 1 Jönköping University, Jönköping, Sweden

The paper describes a survey study in which both quantitative and qualitative data are collected regarding the perceived wearability and appeal of eight different light logging devices, as well as their effect on positioning on the human body.

 The study responds to and expands the ongoing discussion regarding the “optimal” location of a wearable light logging device, where a trade-off must be made between the reliability and accuracy of the exposure data, the wearing comfort and usability, and the wearer’s appearance and acceptability. The mixed-methods approach combines quantitative survey data with descriptive information about experiences and opinions to offer a more comprehensive, credible, and insightful understanding of the wearables research topic.

 The paper presents its results in a highly comprehensive manner, with clear documentation and open availability of the applied methodology, reflecting a deliberate and commendable intention to ensure transparency and reproducibility.

 The following suggestions might enhance overall motivation and reporting of results.

 The **Title** may be slightly misleading because the participants have not been wearing the actual light logger, so ‘wearability’ might not be entirely suitable. In the abstract, the term ‘perceived wearability' is used, which better captures the experience. In the conclusion, ‘placement locations’ is mentioned, which accurately captures the actual study content.

 In the **Background**, the authors correctly mention that wearable light loggers are becoming key tools in, for example, circadian, sleep, and myopia research. However, the discussion about wearables extends beyond simple light (‘illuminance’) logging, especially since recording spectral power distributions requires more advanced devices that often need more space. It may be good to comment on that and perhaps reflect on it in the Discussion (broadening the part already mentioned under ‘Use of subjective measures’).

 In the opening lines of the **Method**, the authors stated that they have developed eight possible designs for wearable loggers. Still, they have only identified potential and actual locations for light logging devices. They mention that some locations and devices correspond to existing, real devices, while others are specifically conceptualised. Would it be possible to provide a more detailed view than just a very abstract (location) image of the actual devices, as presented in Figure 1?

 Working with a questionnaire has the advantage of reaching a large population worldwide, but – in this case – the significant disadvantage is that the actual devices are not physically worn, experienced, and tested. An explanation of why the devices or placebo versions have not been physically worn by (a sample of the population) would strengthen the motivation for the chosen methodology. See also, for example, Aarts *et al*. (2017).

 Regarding **Results**, what was the reasoning behind making the distinction between respondents from the USA, the UK, and the rest of the world? (How) did the recruitment procedure influence the skewed and increased participation from the USA and UK, in particular, since the authors are based in Europe? Since even the Conclusions reflect on the absence of geographical variations, was there a reason to expect a difference?

 Was it known who already has experience wearing glasses or wrist-worn trackers (such as FitBit or Apple Watch) in their everyday life?  For example, the ‘Glasses’ scored lowest in attractiveness/acceptability rankings. Generally, glasses have a significant effect on a person's appearance, which may explain their ranking position. Respondents may not have realised that, in some cases, the light logging can be attached to existing glasses.

 For the Qualitative results, a slightly broader and more elaborate description of the findings would enhance the article. In particular, towards the end, the results are currently largely quotations from the respondents rather than quotations supporting the reported results.

 Since the presented designs appear to be somewhat lacking in detail, how reliable would concerns about appearance be? Respondents are quoted as referring to the devices as ‘ugly’ and ‘medical equipment’, but this appears rather difficult to distinguish from the images presented in Figures 1 and 2 (2D illustrations).

 The **Discussion** can benefit from the lack of actual wearing experience (as mentioned before), but also from the fact that, despite the wearing comfort and appearance, there are still risks of losses and forgetfulness. Some devices, such as the chest or head pin, may remain on the fabric and either end up in the laundry or be forgotten to charge.

Is the study design appropriate and does the work have academic merit?

Yes

Is the work clearly and accurately presented and does it cite the current literature?

Yes

If applicable, is the statistical analysis and its interpretation appropriate?

Yes

Are all the source data underlying the results available to ensure full reproducibility?

Yes

Are the conclusions drawn adequately supported by the results?

Yes

Are sufficient details of methods and analysis provided to allow replication by others?

Yes

Reviewer Expertise:

Lighting Science and Technology, Building Physics and Environmental Design, Human Factors in Lighting

I confirm that I have read this submission and believe that I have an appropriate level of expertise to confirm that it is of an acceptable scientific standard.

## References

[1] : Performance of personally worn dosimeters to study non-image forming effects of light: Assessment methods. *Building and Environment* .2017;117: 10.1016/j.buildenv.2017.03.002 60-72 10.1016/j.buildenv.2017.03.002

# Reviewer report: Reviewer response for version 1

**DOI:** 10.21956/openreseurope.22703.r61078

**Published:** 2025-10-01

**Authors:** Dorukalp Durmus

**Affiliations:** 1 The Pennsylvania State University, University Park, Pennsylvania, USA

The study is very timely, especially considering the increasing interest in wearable dosimeters to collect light exposure data in the wild. User acceptability is indeed important for collecting long-term dosimeter data reliably in the field. The study is novel, data collection and statistical analyses seem robust. Results (highest preference for chest pin followed by wrist and necklace compared to the lowest preference for glasses and hat pin) are not surprising, but it is good to have some insight into user perspectives.

 I think the main novelty of this study lies in the identification of participants’ resistance for wearable dosimeter positions where light exposure to the eye can be most accurately replicated (i.e., glasses). The qualitative part of the survey seems to raise two major but reasonable concerns brough up by the participants: 1) practical use 2) aesthetics. It is good that the authors discussed potential methods to address these challenges.

 The below are suggestions that can improve the article.

 Limitations of the study should be discussed. Participants’ responses are likely impacted by their previous exposure to similar technology. Therefore, their actual use of a dosimeter in these locations could vary from their hypothetical/predicted responses (e.g., participants might find that their concern over physical discomfort is overestimated, but other unforeseen problems may arise). Adding screening questions on participants’ attitudes and beliefs against technology (Do they use smart watches? Are they technophobe vs technophile? etc) could have helped sort out these convictions. Another important limitation (true for most surveys) is the selection bias. People who are willing to complete such a survey is likely interested in the idea of using a wearable dosimeter in the first place. Finally, “I don’t know/I don’t care” response in the surveys would have increased data reliability.

 Figure 1: I assume the red dot represents the median, but it should be explained in the figure caption. I don’t think the red dots should be connected with the dotted lines since the categories (dosimeter position) is not continuous.

 All constants and variables, including n and p of p-value, should be italicized.

 Table 1: The title for age should be “Median age (min-max)” to match the data in the columns, assuming that is the median age.

Is the study design appropriate and does the work have academic merit?

Yes

Is the work clearly and accurately presented and does it cite the current literature?

Yes

If applicable, is the statistical analysis and its interpretation appropriate?

Yes

Are all the source data underlying the results available to ensure full reproducibility?

Yes

Are the conclusions drawn adequately supported by the results?

Yes

Are sufficient details of methods and analysis provided to allow replication by others?

Yes

Reviewer Expertise:

Human factors, colorimetry, lighting, light pollution

I confirm that I have read this submission and believe that I have an appropriate level of expertise to confirm that it is of an acceptable scientific standard.
